# Nursing intervention for improving self-esteem and coping patterns among adolescents with depressive and anxiety disorders

**DOI:** 10.1186/s40359-026-05345-0

**Published:** 2026-08-21

**Authors:** Salma Bakry Attia, Ghada Mohamed Mourad, Hoda Sayed Mohamed, Asmaa Mohamed Khalifa

**Affiliations:** Department of Psychiatric and Mental Health Nursing, Faculty of Nursing, Ain Shams University, Cairo, Egypt

**Keywords:** Adolescents, Anxiety disorders, Coping patterns, Depressive disorders, Nursing intervention, Self-esteem

## Abstract

**Background:**

Adolescents with depressive and anxiety disorders frequently experience diminished self-esteem and maladaptive coping, highlighting the need for targeted nursing interventions to enhance self-perception and foster adaptive coping in this population.

**Aim:**

The study aimed to examine changes observed following the intervention delivered as part of routine clinical care for adolescents with depressive and anxiety disorders on their self-esteem and coping patterns.

**Methods:**

A quasi-experimental one-group pre- and post-test design was used with seventy adolescents with depressive and anxiety disorders. Data were collected using an interviewing questionnaire (socio-demographic characteristics and psychiatric and physical history), the Rosenberg Self-Esteem Scale (RSES), and the Adolescent-Coping Orientation for Problem Experiences (A-COPE). The intervention program focused on enhancing self-esteem and fostering adaptive coping patterns. Data were analyzed using Wilcoxon signed-rank tests and Spearman’s correlation.

**Results:**

Statistically significant changes in median scores for self-esteem and coping domains were observed following implementation of the nursing intervention. Significant positive correlations between self-esteem and coping patterns pre- and post-intervention were also observed.

**Conclusion:**

While the quasi-experimental one-group pre- and post-test design limits causal inferences, the observed improvements in self-esteem and coping patterns suggest that the nursing intervention may benefit adolescents with depressive and anxiety disorders. These findings support incorporating such interventions into mental health services and highlight the importance of future randomized controlled trials to confirm its effectiveness.

**Trial registration:**

The study was retrospectively registered with the Pan African Clinical Trial Registry (PACTR) (PACTR 202508828331881) on 21 August 2025.

## Introduction

Adolescence is a critical developmental stage characterized by rapid biological, psychological, and social changes, making young people vulnerable to mental health problems [1]. Depressive and anxiety disorders are the most common conditions among adolescents and are major causes of psychosocial impairment worldwide [2, 3], affecting identity formation, academic performance, and peer relationships [4]. The prevalence of anxiety and depression among children and adolescents in low- and middle-income countries ranges from 1% to 58% for depression and 1% to 30% for anxiety [5]. In Egypt, prevalence rates among adolescents are 4.9% for depressive disorders and 6.8% for anxiety disorders, with slightly higher rates among adolescent males [6]. Depression results from a complex interaction of social, psychological, and biological factors. It is defined by persistent sadness and loss of interest, both of which significantly impair daily functioning, including sleep, appetite, energy, and cognition, whereas anxiety is characterized by excessive fear and apprehension [7]. A crucial psychosocial factor influencing adolescent mental health is self-esteem, which serves as a central determinant of well-being and strongly affects motivation, resilience, and social interactions [8]. Evidence consistently shows that diminished self-esteem is linked to greater risk of depression and anxiety, creating a vicious cycle in which negative self-concept reinforces depressive and anxious symptoms [9, 10].

Given this close association, it is also important to consider the role of coping in shaping adolescents’ psychological adjustment. Closely linked to self-esteem are coping patterns, which refer to the cognitive and behavioral efforts individuals use to manage stressful life events [11]. During adolescence, the use of adaptive coping such as problem-solving, seeking support, or positive reframing has been shown to buffer against stress and improve psychological adjustment [12]. In contrast, reliance on maladaptive or avoidant coping, such as rumination or withdrawal, is strongly linked to increased vulnerability to depression and anxiety [13, 14].

Given the long-term effects of depression and anxiety, early and structured interventions are essential. Nursing interventions have emerged as effective approaches in addressing these challenges by simultaneously enhancing self-esteem and promoting adaptive coping patterns. Nurses, through their holistic and patient-centered orientation, are uniquely positioned to deliver psycho-education, counseling, and skills training tailored to the developmental needs of adolescents.

Evidence from recent intervention studies demonstrates that nurse-led programs can reduce symptom severity, improve self-esteem, and foster healthier coping behaviors. Although evidence suggests potential benefits of nurse-led interventions for adolescents with depression and anxiety, further research is needed to better understand their effects on these outcomes [15, 16]. Such an approach contributes to evidence-based nursing practice and supports the integration of nurses as key providers in adolescent mental health care [17].

Accordingly, the present study aims to explore changes in self-esteem and coping patterns following a nursing intervention delivered within routine clinical care and to generate hypotheses for future controlled studies.

### Hypothesis

This study hypothesized that the nursing intervention would enhance self-esteem and promote the adoption of more adaptive coping patterns among adolescents with depressive and anxiety disorders.

## Materials and methods

### Study design and setting

A quasi-experimental one-group pre- and post-test design was used. The study was conducted at the Neuropsychiatric Outpatient Clinic for Children and Adolescents at El-Khanka Mental Health Hospital, and the Adolescent Outpatient Clinic at El-Abbassiya Mental Health Hospital affiliated with the General Secretariat of Mental Health and Addiction Treatment, and the Egyptian Ministry of Health and Population. This design was chosen to assess within-group changes resulting from the nursing intervention.

The same intervention protocol (session structure and educational materials) was used at both study sites to ensure consistency in program delivery.

### Study subjects

A purposive sample of 70 adolescents diagnosed with depressive and/or anxiety disorders was recruited from the aforementioned outpatient settings. A pilot study was initially conducted with 30 adolescents to test the clarity and feasibility of the study instruments and procedures. The pilot results indicated that all items were clear and understandable, and no modifications were required. Therefore, the pilot participants were retained and included in the final study sample to maximize the available sample size.

Inclusion and exclusion criteria were applied to ensure the appropriateness of the selected participants, as follows:

### Inclusion criteria

- Adolescents aged 13–18 years.
- Both male and female participants.
- Adolescents with a confirmed diagnosis of depressive and/or anxiety disorders.
- Willing to participate in the study.

### Exclusion criteria

- Adolescents with severe cognitive, sensory, or physical disabilities that prevented active participation in the intervention sessions.

### Age justification

Although the World Health Organization (WHO) defines adolescence as the age range of 10–19 years, the present study intentionally targeted participants aged 13–18 years. This decision was made to ensure sufficient cognitive and emotional maturity to engage effectively with the intervention components. The intervention involved activities such as thought recording, emotion wheel exercises, problem-solving tasks, and time management strategies, all of which require a level of abstract thinking, self-awareness, and communication skills that typically emerge from early adolescence onward.

Within the context of mental health services in Egypt, individuals aged 19 years are generally classified and treated as adults in outpatient psychiatric clinics. Including them could have introduced variability in terms of clinical settings, therapeutic approaches, and developmental stages. Therefore, restricting the sample to adolescents aged 13–18 years enhanced sample consistency and ensured the intervention’s relevance and applicability to the adolescent population.

### Sample size

An a priori power analysis was conducted using G*Power 3.1.9.7 [18] for a two-tailed paired t-test with α = 0.05, desired power = 0.95, and a medium effect size (dz = 0.50). The analysis indicated that a minimum of 54 participants was required. To increase the precision of the findings and to account for possible attrition or missing data, a total of 70 adolescents were recruited, thus exceeding the minimum required sample size.

### Measures

Three tools were used to conduct the study:

### Tool I: interviewing questionnaire

The researcher developed this tool in Arabic, which included two parts:

- *Part I: Socio-Demographic Characteristics of Adolescents*, including age, sex, educational level, residence, birth order, and monthly family income.
- *Part II: Psychiatric and Physical History*, which addressed the type of psychiatric disorder (specifically depressive disorders classified under mood disorders and anxiety disorders classified under anxiety and fear-related disorders according to DSM-5 and ICD-11), duration since diagnosis, type of management received, presence of chronic medical conditions (if any) and their classification, and family history of psychiatric illness.

### Tool II: Rosenberg Self-Esteem Scale (RSES)

The Rosenberg Self-Esteem Scale (RSES) was originally developed by Rosenberg [19] and translated into Arabic and linguistically refined by a language and translation specialist to ensure clarity and comprehension for adolescents. It was used to assess both positive and negative self-perceptions. The scale consists of 10 items, with 5 positively worded statements (e.g., “I take a positive attitude toward myself”) and 5 negatively worded statements (e.g., “At times I think I am no good at all”). The Arabic version was back-translated to ensure accuracy.

### Scoring system

Responses are rated on a 4-point Likert scale ranging from 1 (strongly disagree) to 4 (strongly agree). Total scores are categorized as follows:

- Low self-esteem = 10–25, moderate self-esteem = 26–29, high self-esteem = 30–40 [20].

### Tool III: Adolescent-Coping Orientation for Problem Experiences (A-COPE)

The Adolescent-Coping Orientation for Problem Experiences (A-COPE) was originally developed by Patterson, J. M., & McCubbin, H. I. (1987) [21], which was translated into Arabic and linguistically refined by a language and translation specialist to ensure that the items were clear and easily understood by adolescents, and then back-translated to ensure accuracy. It was employed to assess the coping strategies that adolescents use to manage and adjust to stressful situations, and comprises 12 subscales as follows:

**Table:** 

No	Subscale	Item Numbers	No of items
Subscale 1:	Ventilating feelings	19*, 22, 26*, 28*, 49*, 51	6
Subscale 2:	Seeking diversions	2, 9, 11, 33, 37, 43, 48, 53	8
Subscale 3:	Developing self-reliance and optimism	15, 25, 32, 40, 45, 47	6
Subscale 4:	Developing social support	4, 14, 18, 30, 35, 52	6
Subscale 5:	Solving family problems	1, 12, 31, 39, 41, 50	6
Subscale 6:	Avoiding problems	8*, 24*, 36, 42*, 46*	5
Subscale 7:	Seeking spiritual support	21, 23, 44	3
Subscale 8:	Investing in close friends	16, 29	2
Subscale 9:	Seeking professional support	6, 34	2
Subscale 10:	Engaging in demanding activity	10, 13, 27, 54	4
Subscale 11:	Being humorous	3, 20	2
Subscale 12:	Relaxing	5, 7*, 17, 38	4

### Scoring system

The scale is a 5-point Likert scale, ranging from 1 (Never) to 5 (Most of the Time) for all items, except for items 7, 8, 19*, 24*, 26*, 28*, 42*, 46*, and 49*, which are reverse-scored, with 1 representing Most of the Time and 5 representing Never.

The total scores were classified as follows:

**Table:** 

Total coping	No of items	Mild Use	Moderate Use	High Use
	54	54–125	126–197	198–270

### Data collection

After obtaining the necessary approvals, the researcher conducted individual interviews with each adolescent, providing a brief explanation of the study’s nature and objectives, and requesting their voluntary participation. All adolescents were informed that participation was entirely voluntary, and consent was obtained from both the adolescents and their caregivers. Data collection began with the interviewing questionnaire (socio-demographic characteristics and psychiatric and physical history), which was completed by the researcher in approximately 10 min per participant. Subsequently, the Rosenberg Self-Esteem Scale (RSES) was administered, taking about 10 min for each adolescent. Finally, the Adolescent-Coping Orientation for Problem Experiences (A-COPE) Scale required approximately 20 min per participant. In total, each adolescent spent around 40 min completing all study instruments.

**Fig. 1:**
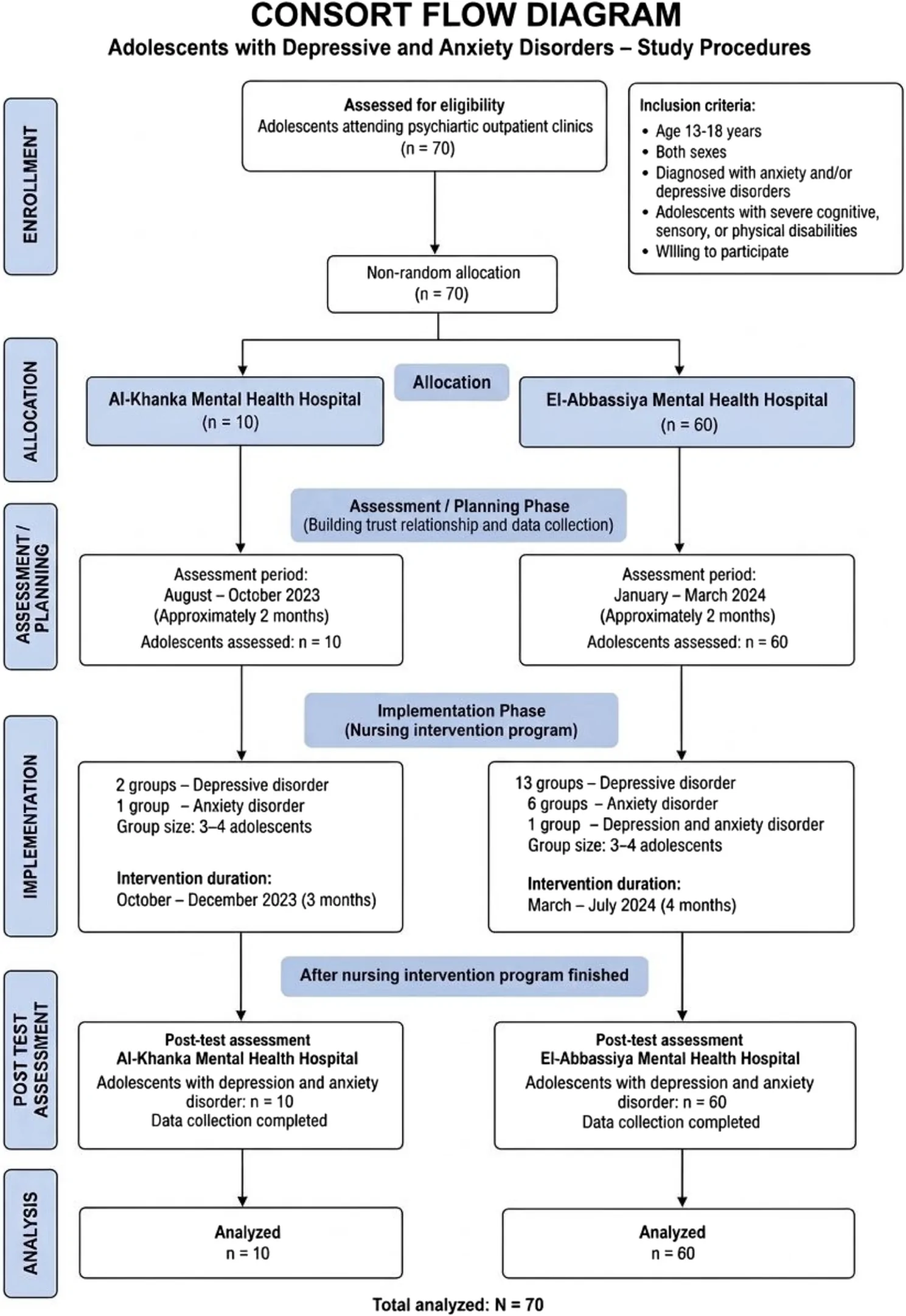
Summary of the data collection process

The study, employing a one-group pre- and post-test interventional design, was conducted over an 11-month period from mid-August 2023 to the end of July 2024. At El-Khanka Mental Health Hospital, data collection was conducted over 2 months (August–October 2023), followed by the implementation of the nursing intervention over 3 months (October–December 2023). Subsequently, at El-Abbassiya Mental Health Hospital, data collection and the intervention were conducted concurrently over a 6-month period (January–July 2024). This structured timeline ensured systematic administration of the intervention and comprehensive data collection across both settings (Fig. 1).

Although the intervention was implemented at two different sites and time periods, the same standardized intervention protocol and session content were applied in both settings to ensure consistency in delivery. Outcomes from both sites were pooled for the main analysis because of the relatively small sample size at each location. Site was not modeled as a clustering variable in the statistical analysis; therefore, potential site-related variance cannot be completely excluded.

### Diagnostic procedures

Psychiatric diagnoses were established by licensed psychiatrists as part of routine clinical care in accordance with the Diagnostic and Statistical Manual of Mental Disorders, Fifth Edition (DSM-5) criteria. Diagnoses were based on comprehensive clinical interviews and standard psychiatric evaluation procedures. Only adolescents with a documented clinical diagnosis in their medical records were eligible for inclusion in the study. Diagnostic eligibility was verified during recruitment through review of the participants’ medical records. No structured diagnostic interview was administered specifically for research purposes, and diagnoses were not independently re-verified for the study. The distribution of diagnostic categories (depressive disorders, anxiety disorders, and comorbid depressive and anxiety disorders) in the sample is presented in Table 2. The sample included adolescents diagnosed with depressive disorders (64.3%), anxiety disorders (30%), or comorbid depressive and anxiety conditions (5.7%).

### Methods of pharmacotherapy

Information regarding current pharmacological treatment was obtained from participants’ medical records, including the presence of antidepressant or anxiolytic medication as part of routine psychiatric management.

The majority of participants (97.1%) were receiving pharmacotherapy as part of routine psychiatric care as presented in Table 2. Treatment consisted primarily of selective serotonin reuptake inhibitors (SSRIs), specifically fluoxetine (20–40 mg/day) and sertraline (50–200 mg/day). Participants had been maintained on stable medication regimens for at least four weeks before baseline assessment, according to their medical records. No medication changes were introduced as part of the study protocol, and medication management was conducted independently by the treating psychiatrists. Because the intervention was delivered in the context of ongoing pharmacological treatment, the observed improvements cannot be attributed exclusively to the nursing intervention, and pharmacotherapy may represent a potential source of confounding.

### Intervention contents

The intervention was primarily guided by Stress and Coping Theory [22], with additional integration of cognitive-behavioral principles. From a nursing perspective, the program is most closely aligned with Orem’s Self-Care Deficit Theory, which supports the development of self-management and adaptive coping among adolescents. Also, the intervention was described in accordance with TIDieR reporting guidelines to ensure transparency and reproducibility “Supplementary File 1 (TIDieR checklist)”.

The structured intervention consisted of 20 weekly sessions, each lasting approximately 60 min, designed to enhance self-esteem and promote adaptive coping patterns among adolescents with depressive and anxiety disorders. The sessions were conducted in small groups consisting of 3–4 adolescents to facilitate interaction, peer support, and active participation.

Prior to implementation, the intervention content was reviewed by experts in psychiatric nursing and psychiatric medicine to ensure its relevance, safety, and appropriateness for adolescents.

The intervention was delivered by the principal researcher, a teaching assistant in the Psychiatric and Mental Health Nursing Department, who has academic training in mental health nursing and experience in implementing educational and supportive interventions for adolescents.

The co-authors supervised the implementation of the intervention and monitored the adherence to the planned session structure to ensure consistency and fidelity of program delivery. The principal researcher received structured training on intervention procedures and session delivery prior to implementation. A standardized session outline was used to maintain consistency across sessions. Intervention fidelity was monitored using a structured session checklist to ensure adherence to the predefined session content and delivery procedures.

Each session followed a standardized structure. Sessions began with a brief greeting and assessment of participants’ motivation, followed by a review of the previous session. The objectives of the new topic were then introduced using simple language appropriate for adolescents’ educational level. Practical exercises and interactive activities were conducted to reinforce learning. Each session concluded with feedback, discussion, and reinforcement of the key concepts.

Various interactive teaching strategies were used to enhance engagement, including group discussions, motivational videos, storytelling, written assignments, demonstrations, role play, and small-group activities. Participants also received a booklet summarizing the intervention content to support learning and encourage the application of new skills in daily life.

The intervention included both theoretical and practical components as presented in Table 1. The theoretical component provided an overview of adolescence, depressive and anxiety disorders, emotional awareness, self-esteem, and coping strategies. The practical component focused on modifying negative thoughts, improving self-esteem, and developing adaptive coping skills through techniques such as goal planning, positive self-talk, gratitude exercises, relaxation techniques, problem-solving strategies, anger management, time management, and communication skills.

**Table 1:** A detailed session-by-session plan

Phase	Session	Topic	Main Content	Techniques / Procedures
Pre-test phase	Session 1	Program introduction and baseline assessment	Introduction to the intervention program and administration of Rosenberg Self-Esteem Scale (RSES) and Adolescent-Coping Orientation for Problem Experiences (A-COPE)	Explanation, structured tool administration
Implementation phase	Session 2	Understanding adolescence and psychological changes	Developmental characteristics, psychological needs, and common challenges during adolescence	Lecture; group discussion
	Sessions 3–4	Understanding depression and anxiety disorder.	Causes, signs, symptoms, and management.	Lecture; discussion
	Session 5	Emotional awareness	Identifying emotions using the emotion wheel and encouraging healthy emotional expression.	Emotion wheel exercise; guided discussion; role play.
	Session 6	Cognitive restructuring	Thought modification technique	Thought-record technique; cognitive reframing
	Sessions 7–10	Building positive Self-Esteem	Understanding concept of self-esteem, factors affecting it, and recognizing signs of low self-esteem. Goal setting, positive self-talk, gratitude practices, and confidence-building activities.	Goal setting; positive self-talk; confidence exercises
	Session 11	Introduction to coping strategies	Meaning of coping and different coping strategies used in stressful situations	lecture; group discussion
	Session 12	Practicing self-care	Practicing healthy self-care behaviors.	Group discussion; self-care planning
	Sessions 13–14	Mind–Body relaxation	Breathing exercises, meditation, yoga, and progressive muscle relaxation	Group discussion; demonstration.
	Session 15	Problem-Solving as a Coping Skill	Problem-solving techniques; applying in different scenarios.	Scenario-based training
	Session 16	Anger management	Strategies for managing anger and controlling emotional reactions in provoked situations.	Role play; behavioral rehearsal; Scenario-based training.
	Session 17	Time management for stress reduction	Developing personal time-management strategies.	Planning exercises
	Session 18	Effective communication and social support	Effective communication techniques and building supportive relationships.	Role play; interaction training
	Session 19	Healthy leisure activities and behavioral activation	Encouraging engagement in positive recreational and social activities	Activity scheduling
Post-test phase	Session 20	Post-test assessment	Re-administration of Rosenberg Self-Esteem Scale (RSES) and A-COPE	Re-assessment of tools

All participants (*n* = 70) completed the intervention sessions. This may be attributed to the structured follow-up within the clinical setting and continuous engagement strategies

In addition to the weekly sessions, the researcher maintained weekly telephone follow-up with participants to monitor progress, address challenges, and reinforce adherence to the intervention strategies. Participants were also encouraged to communicate through WhatsApp groups to promote peer support, share experiences, and maintain motivation throughout the program.

### Statistical analysis

Data were analyzed using the Statistical Package for the Social Sciences (SPSS), version 27. Descriptive statistics were presented as numbers, percentages, means, standard deviations, medians, and interquartile ranges, as appropriate. The normality of the data distribution was assessed using the Shapiro–Wilk test, which indicated that the data were not normally distributed (*p* < 0.05). Because the normality assumption was not satisfied, non-parametric statistical tests were used. Consequently, the Wilcoxon signed-rank test was employed to compare pre- and post-intervention scores of self-esteem and coping skills. Effect sizes (r) were calculated from the standardized Wilcoxon signed-rank test statistic using the formula r = Z/√N, where Z represents the standardized test statistic and N represents the number of non-zero paired observations included in the analysis [23]. The 95% confidence intervals (95% CIs) for effect sizes were estimated using bias-corrected and accelerated (BCa) bootstrap resampling with 100,000 bootstrap replicates implemented in R (version 4.6.1) [24]. Additionally, Spearman’s rank correlation coefficient (ρ) was applied to examine the associations between self-esteem and coping skills. A p-value < 0.05 was considered statistically significant.

### Validity

The standardized Arabic version of the A-COPE was used without modification to preserve its original conceptual structure. However, confirmatory factor analysis was not conducted in the present study.

Five experts evaluated each item for cultural and linguistic relevance using a 4-point scale. The item-level content validity index (I-CVI) ranged from 0.80 to 1.00 for all items, exceeding the recommended minimum of 0.78 for a panel of this size [25]. At the scale level, the Rosenberg Self-Esteem Scale achieved S-CVI/Ave = 1.00 and S-CVI/UA = 1.00, indicating perfect agreement among experts. The Adolescent-Coping Orientation for Problem Experiences Scale demonstrated S-CVI/Ave = 0.96 and S-CVI/UA = 0.81, surpassing the accepted cut-off values (S-CVI/Ave ≥ 0.90; S-CVI/UA ≥ 0.80) and confirming excellent overall content validity. These results indicate that all items are culturally appropriate and conceptually equivalent to the originals, providing strong support for their use in the main study.

### Test–retest reliability

Test–retest reliability was assessed over a two-week interval using the same participants. The results demonstrated good temporal stability for the total self-esteem score (Spearman’s ρ = 0.681, *p* < 0.001) and excellent temporal stability for the coping total score (Pearson’s *r* = 0.938, *p* < 0.001), indicating that both instruments produced consistent and reliable measurements over time.

#### Internal consistency

Internal consistency reliability was evaluated using Cronbach’s alpha coefficient. The Rosenberg Self-Esteem Scale (RSES) showed good internal consistency (α = 0.887), while the A-COPE scale demonstrated acceptable internal consistency (α = 0.850). Subscale-level reliability coefficients showed variability, with several subscales demonstrating acceptable to good reliability (e.g., Developing Self-Reliance and Optimism α = 0.678; Avoiding Problems α = 0.502; Being Humorous α = 0.702), while others showed lower internal consistency. This variability is consistent with the original development study of the A-COPE, which reported Cronbach’s alpha values ranging from 0.50 to 0.76 across subscales. Subscales with fewer items and those reflecting heterogeneous coping behaviors tended to yield lower reliability estimates. Therefore, subscale-level findings were interpreted with caution, and greater emphasis was placed on total coping scores.

## Results

### Part I: socio-demographic characteristics

Table 2 presents the socio-demographic characteristics of adolescents included in the study. The largest proportion was aged 17–18 years (38.6%), and 60% were female. In terms of education, most participants completed primary education (52.9%). The majority of participants resided in urban areas (90%), and a considerable proportion were the eldest sibling in the family (60%). Additionally, over half of the adolescents reported that their monthly family income adequately met living and medical needs (57.1%).

**Table 2:** Frequency distribution of the studied adolescents according to socio-demographic characteristics (*n* = 70)

Socio-Demographic Characteristics	No.	%
Age (years)	18	25.7
13–<15		
15–<17	25	35.7
17–18	27	38.6
*Mean ± SD*	15.67 ± 1.43	
Sex	28	40.0
Male		
Female	42	60.0
Educational Level	1	1.4
Illiterate		
Reads and writes	28	40.0
Primary education	37	52.9
Secondary education	4	5.7
Residence	63	90
Urban		
Rural	7	10.0
Birth Order	42	60.0
Eldest brother/sister		
Second brother/sister	20	28.6
Youngest brother/sister	8	11.4
Only child	0	0.0
Monthly Family Income	40	57.1
Enough for living and medical needs		
Not enough for living and medical needs	30	42.9

#### Part II: adolescent’s psychiatric and physical history

Table 3 shows that the majority of adolescents were diagnosed with depression (64.3%) or anxiety (30%). Most participants had been diagnosed for less than 6 months (44.3%), and the predominant treatment was pharmacological (85.7%). The majority did not report chronic medical conditions (88.6%), and slightly more than half had no family history of psychiatric illness (52.9%).

**Table 3:** Distribution of the studied adolescents according to psychiatric and physical history (*n* = 70)

Psychiatric and Physical History	No.	%
Type of Psychiatric Disorder	45	64.3
Depression		
Anxiety	21	30.0
Both	4	5.7
Duration Since Diagnosis (months)	31	44.3
Less than 6 months		
6–12 months	17	24.3
> 12 months	22	31.4
*Mean ± SD*	11.30 ± 3.74	
Type of Management Received	60	85.7
Pharmacological		
Non-pharmacological (Psychotherapy)	2	2.9
Both	8	11.4
Presence of Chronic Medical Conditions	62	88.6
No		
Yes (Type)	8	11.4
Backache	1	12.5
Brain tumor	1	12.5
Epilepsy	2	25.0
Fibromyalgia	1	12.5
Headache	1	12.5
Ichthyosis	1	12.5
Stomach ulcer	1	12.5
Family History of Psychiatric Illness	37	52.9
None		
Yes	33	47.1
Depression	14	20.0
Anxiety	7	10.0
Other	12	17.1

Table 4 shows a significant positive correlation between self-esteem and total coping patterns pre-intervention (ρ = 0.343, *p* = 0.004) and post-intervention (ρ = 0.390, *p* = 0.001), indicating that higher self-esteem was consistently associated with better coping abilities.

**Table 4:** Comparison of self-esteem scores pre- and post-intervention among adolescents (*n* = 70)

Variable	Pre-intervention	Post-intervention	Z	*p*-value	Effect size (*r*)	95% BCa CI	
	Median (IQR)	Median (IQR)				Lower	Upper
Self-Esteem	21.5 (20–25)	30 (29–31)	-7.29	< 0.001	0.87	0.8705	0.8722

According to Cohen [26], *r* = 0.1 indicates a small effect, *r* = 0.3 a medium effect, and *r* = 0.5 a large effect

#### Part III: self-esteem in adolescents

Table 5 shows statistically significant changes between the median self-esteem pre- and post-intervention. The median self-esteem increased from 21.5 (IQR 20–25) pre-intervention to 30 (IQR 29–31) post-intervention. The change was statistically significant (Z = -7.29, *p* < 0.001) with a large effect size (*r* = 0.87), indicating a substantial observed within-group change following the intervention.

**Table 5:** Comparison of total coping pattern scores pre- and post-intervention among adolescents using Wilcoxon Signed-Rank Test (*n* = 70)

Variable	Pre-intervention	Post-intervention	Z	*p*-value	Effect size (*r*)	95% BCa CI		Significant after Bonferroni (*p* < 0.004)
	Median (IQR)	Median (IQR)				Lower	Upper	
Total Coping Pattern	112 (103–122)	118 (111–131)	-5.83	< 0.001	0.71	0.5403	0.8011	Yes

Table 6 shows a statistically significant improvement in the total coping score following the intervention (*p* < 0.001), which remained significant after Bonferroni correction (adjusted significance level *p* < 0.004).

**Table 6:** Correlation between self-esteem and total coping pattern scores pre- and post-intervention among adolescents (*n* = 70)

The study variables			Pre-intervention	Post-intervention
			Self-Esteem	Self-Esteem
Pre-intervention	Coping pattern	(ρ)	0.343^**^	
		*p* value	0.004	
Post-intervention	Coping pattern	(ρ)		0.390^**^
		*p* value		0.001

(ρ) = Spearman’s rank correlation coefficient

** Correlation is significant at the 0.01 level (two-tailed)

## Discussion

The present study employed a one-group pre–post design without a control or comparative group; therefore, causal inferences regarding the effectiveness of the intervention cannot be established. Although statistically significant improvements were observed, the study did not assess clinical remission, symptom severity, or functional outcomes; therefore, clinical significance cannot be determined.

The observed changes may be influenced by multiple alternative explanations, including maturation, regression to the mean, testing effects, Hawthorne effects, natural course of symptoms, and the concurrent use of pharmacological treatment, which was received by most participants. In addition, several uncontrolled contextual factors may have contributed to the observed improvements, such as therapist–participant contact, peer interaction during group sessions, repeated attention associated with the researchers’ assessments, and natural symptom fluctuation over time.

Adolescence represents a critical developmental period characterized by increased vulnerability to depression and anxiety, conditions that are frequently associated with low self-esteem and maladaptive coping strategies [27]. In this context, nursing interventions may provide structured psychosocial support aimed at enhancing self-esteem, improving coping skills, and facilitating psychological adjustment [28]. However, cultural and contextual factors may also shape adolescents’ coping behaviors and help-seeking patterns, potentially influencing engagement with the intervention and limiting the generalizability of the findings beyond the local setting.

Regarding socio-demographic characteristics, most participants were in mid-to-late adolescence, a stage associated with heightened emotional and psychological challenges. Females represented a slightly higher proportion than males in the study sample, reflecting the increased vulnerability of adolescent girls to psychological and psychiatric problems. Most participants were enrolled in basic education and resided in urban areas, which may influence access to services and exposure to stressors. In addition, family dynamics also played a role, as many participants were eldest children, a position often linked with greater responsibility and pressure within the family, which may contribute to increased psychological burden and reduced coping resources.

Regarding adolescents’ psychiatric and physical history, participants were diagnosed with depressive disorders, anxiety disorders, or both. The inclusion of comorbid cases reflects the high co-occurrence of these conditions during adolescence and their shared cognitive and emotional features, including negative thinking patterns, low self-esteem, and maladaptive coping. The intervention was therefore designed to target these common mechanisms rather than disorder-specific symptoms. Depression was the most frequently reported diagnosis, followed by anxiety, while pharmacological treatment was the predominant management approach. These findings are broadly consistent with previous literature reporting increasing rates of adolescent depression and the widespread use of pharmacotherapy in clinical practice [29, 30].

A significant improvement in self-esteem was observed following the intervention. However, the magnitude of change should be interpreted cautiously given the absence of a control group, potential regression to the mean, and measurement reactivity associated with repeated assessments. In addition, the observed redistribution across self-esteem categories should be interpreted as a shift in score distribution rather than a definitive transition between clinically distinct levels.

This is consistent with previous evidence suggesting that structured psychosocial and nursing interventions may support adolescents’ self-esteem [31–33]. However, cultural and social factors may have influenced adolescents’ coping and help-seeking behaviors, potentially affecting engagement with the intervention and limiting the generalizability of the findings.

Similarly, a significant improvement in total coping scores was observed following the intervention. These results align with prior studies suggesting that cognitive-behavioral, psycho-educational, and activity-based interventions may be associated with improvements in coping patterns among adolescents [34, 35].

Although statistically significant improvements were observed, the clinical relevance of these changes remains uncertain in the absence of a control group and standardized measures of symptom severity. Therefore, the findings should be interpreted as preliminary and hypothesis-generating rather than confirmatory evidence of intervention effectiveness.

Finally, a positive association was found between self-esteem and coping strategies, indicating that higher self-esteem scores were associated with greater use of adaptive coping patterns. This finding is consistent with previous studies suggesting that self-esteem plays an important role in shaping coping behaviors [36–39]. However, another study has reported no significant association, suggesting that this relationship may vary depending on contextual and methodological factors [40].

Overall, the findings should be interpreted with caution due to methodological limitations of the study, and further controlled studies are warranted to confirm these preliminary findings.

### Limitations

This study has several limitations. The single-group pre-test–post-test design without a control group limits causal inference, and the observed changes may have been influenced by concurrent pharmacological treatment and other uncontrolled factors. In addition, the study was retrospectively registered; pilot participants were included in the final sample. Therefore, the findings should be considered preliminary, and further randomized controlled studies are needed to confirm these results.

## Conclusion

Although the lack of a control group limits causal inference, improvements in self-esteem and coping patterns were observed following participation in the intervention among adolescents with depressive and anxiety disorders. These findings provide quasi-experimental one-group pre- and post-test design evidence suggesting that nurse-led interventions may have potential benefits in supporting adolescents’ psychological and social well-being. However, given the study design, comparative effectiveness cannot be established. Further controlled studies are needed to confirm these findings and strengthen causal inference.

### Implications

The findings indicate that improvements in adolescents’ self-esteem and adaptive coping were observed following participation in the intervention, suggesting that structured psychosocial programs may represent a promising approach for supporting adolescents with depressive and anxiety disorders in community settings. However, given the study design, causal conclusions cannot be drawn. Future randomized controlled trials with larger samples are needed to evaluate the effectiveness of the intervention and strengthen causal inference. Further research should also examine outcomes separately among adolescents with depressive and anxiety disorders and assess the long-term sustainability of observed changes.

## Data Availability

The data supporting the findings of this study are contained within the submitted manuscript. No additional datasets are available.

## Abbreviations

A-COPE: Adolescent-Coping Orientation for Problem Experiences
BCa: Bias-Corrected and Accelerated (bootstrap)
CI: Confidence Interval
DSM-5: Diagnostic and Statistical Manual of Mental Disorders, Fifth Edition
ICD-11: International Classification of Diseases, 11th Revision
I-CVI: Item-Level Content Validity Index
IQR: Interquartile Range
PACTR: Pan African Clinical Trial Registry
R: Effect size (correlation coefficient)
RSES: Rosenberg Self-Esteem Scale
SD: Standard Deviation
S-CVI/Ave: Scale-Level Content Validity Index, Averaging Method
S-CVI/UA: Scale-Level Content Validity Index, Universal Agreement
SPSS: Statistical Package for the Social Sciences
SSRIs: Selective Serotonin Reuptake Inhibitors
TIDieR: Template for Intervention Description and Replication
WHO: World Health Organization

## References

[1] Lin J, Guo W. The research on risk factors for adolescents’ mental health. Behav Sci (Basel). 2024;14(4):263. 10.3390/bs14040263. 38667059 10.3390/bs14040263 PMC11047495

[2] de Castro F, Cappa C, Madans J. Anxiety and depression signs among adolescents in 26 low- and middle-income countries: prevalence and association with functional difficulties. J Adolesc Health. 2023;72(1 Suppl):S79–87. 10.1016/j.jadohealth.2022.03.022. 36528385 10.1016/j.jadohealth.2022.11.161 PMC9935490

[3] Polanczyk GV, Salum GA, Sugaya LS, Caye A, Rohde LA. Annual research review: a meta-analysis of the worldwide prevalence of mental disorders in children and adolescents. J Child Psychol Psychiatry. 2015;56(3):345–65. 10.1111/jcpp.12381. 25649325 10.1111/jcpp.12381

[4] Bi Y, Moon M, Shin M. The longitudinal effects of depression on academic performance in Chinese adolescents via peer relationships: the moderating effect of gender and physical activity. Int J Environ Res Public Health. 2022;20(1):181. 10.3390/ijerph20010181. 36612503 10.3390/ijerph20010181 PMC9820040

[5] Ramdhonee-Dowlot K, Balloo K, Morgül E, Essau CA. Prevalence of anxiety and depression among children and adolescents in low- and middle-income countries: a systematic review. Psychiatr Res Clin Pract. 2025;7(4):218–43. 10.1176/appi.prcp.20250026. 41415522 10.1176/appi.prcp.20250026 PMC12710537

[6] Eldeeb MMA. Prevalence of psychiatric disorders in male adolescents in urban areas in Egypt. Al-Azhar Med J. 2021;50(4):3181–92. 10.21608/amj.2021.198398.

[7] Yang H, Wang L, Cao C, Cao X, Fang R, Zhang J, et al. The underlying dimensions of DSM-5 PTSD symptoms and their relations with anxiety and depression in a sample of adolescents exposed to an explosion accident. Eur J Psychotraumatol. 2017;8(1):1272789. 10.1080/20008198.2016.1272789. 28326161 10.1080/20008198.2016.1272789 PMC5328312

[8] Orth U, Robins RW. The development of self-esteem. Curr Dir Psychol Sci. 2014;23(5):381–7. 10.1177/0963721414547414.

[9] Masselink M, Van Roekel E, Oldehinkel AJ. Self-esteem in early adolescence as predictor of depressive symptoms in late adolescence and early adulthood: the mediating role of motivational and social factors. J Youth Adolesc. 2018;47(5):932–46. 10.1007/s10964-017-0727-z. 28785953 10.1007/s10964-017-0727-z PMC5878202

[10] Sowislo JF, Orth U. Does low self-esteem predict depression and anxiety? A meta-analysis of longitudinal studies. Psychol Bull. 2013;139(1):213–40. 10.1037/a0028931. 22730921 10.1037/a0028931

[11] Compas BE, Jaser SS, Bettis AH, Watson KH, Gruhn MA, Dunbar JP, et al. Coping, emotion regulation, and psychopathology in childhood and adolescence: a meta-analysis and narrative review. Psychol Bull. 2017;143(9):939–91. 10.1037/bul0000110. 28616996 10.1037/bul0000110 PMC7310319

[12] Garnefski N, Kraaij V. Specificity of relations between adolescents’ cognitive emotion regulation strategies and symptoms of depression and anxiety. Cogn Emot. 2018;32(7):1401–8. 10.1080/02699931.2016.1232698. 27648495 10.1080/02699931.2016.1232698

[13] Herman-Stahl M, Petersen AC. The protective role of coping and social resources for depressive symptoms among young adolescents. J Youth Adolesc. 1996;25(6):733–53. 10.1007/BF01537451.

[14] Nolen-Hoeksema S, Wisco BE, Lyubomirsky S. Rethinking rumination. Perspect Psychol Sci. 2008;3(5):400–24. 10.1111/j.1745-6924.2008.00088.x. 26158958 10.1111/j.1745-6924.2008.00088.x

[15] Arnold JL, Baker C. The role of mental health nurses in supporting young people’s mental health: a review of the literature. Ment Health Rev J. 2018;23(3):197–220. 10.1108/MHRJ-09-2017-0039.

[16] Kunikata H, Yoshinaga N, Shiraishi Y, Okada Y. Nurse-led cognitive-behavioral group therapy for recovery of self-esteem in patients with mental disorders: a pilot study. Jpn J Nurs Sci. 2016;13(3):355–64. 10.1111/jjns.12114. 26782776 10.1111/jjns.12114

[17] Kumar A, Kearney A, Hoskins K, Iyengar A. The role of psychiatric mental health nurse practitioners in improving mental and behavioral health care delivery for children and adolescents in multiple settings. Arch Psychiatr Nurs. 2020;34(5):275–80. 10.1016/j.apnu.2020.07.022. 33032746 10.1016/j.apnu.2020.07.022 PMC7547148

[18] Faul F, Erdfelder E, Lang AG, Buchner A. G*Power 3: a flexible statistical power analysis program for the social, behavioral, and biomedical sciences. Behav Res Methods. 2007;39(2):175–91. 10.3758/bf03193146. 17695343 10.3758/bf03193146

[19] Rosenberg M. Society and the adolescent self-image. Princeton (NJ). Princeton University Press; 1965. https://www.jstor.org/stable/j.ctt183pjjh.

[20] Acosta García J, Checa y Olmos F, Lucas Matheu M, Parrón Carreño T. Self-esteem levels vs global scores on the Rosenberg self-esteem scale. Heliyon. 2019;5(3):e01378. 10.1016/j.heliyon.2019.e01378. 30963120 10.1016/j.heliyon.2019.e01378 PMC6434180

[21] Patterson JM, McCubbin HI. Adolescent coping style and behaviors: conceptualization and measurement. J Adolesc. 1987;10(2):163–. 10.1016/S0140-1971(87)80086-6. 86. 3611466 10.1016/s0140-1971(87)80086-6

[22] Lazarus RS, Folkman S. Transactional theory and research on emotions and coping. Eur J Pers. 1987;1(3):141–69.

[23] Field A. Discovering statistics using IBM SPSS Statistics. 5th ed. London: Sage; 2018.

[24] Davison AC, Hinkley DV. Bootstrap methods and their application. Cambridge: Cambridge University Press; 1997.

[25] Polit DF, Beck CT. The content validity index: are you sure you know what’s being reported? Critique and recommendations. Res Nurs Health. 2006;29(5):489–97. 10.1002/nur.20147. 16977646 10.1002/nur.20147

[26] Cohen J. Statistical power analysis for the behavioral sciences. 2nd ed. London: Routledge; 1988. 10.4324/9780203771587.

[27] Young KS, Sandman CF, Craske MG. Positive and negative emotion regulation in adolescence: links to anxiety and depression. Brain Sci. 2019;9(4):76. 10.3390/brainsci9040076. 30934877 10.3390/brainsci9040076 PMC6523365

[28] Li Z, Li J, Kong J, Li Z, Wang R, Jiang F. Adolescent mental health interventions: a narrative review of the positive effects of physical activity and implementation strategies. Front Psychol. 2024;15:1433698. 10.3389/fpsyg.2024.1433698. 38993342 10.3389/fpsyg.2024.1433698 PMC11236730

[29] Adams JS, Chien AT, Wisk LE. Mental illness among youth with chronic physical conditions. Pediatrics. 2019;144(1):e20181819. 10.1542/peds.2018-1819. 31201229 10.1542/peds.2018-1819 PMC12930273

[30] Correll CU, Cortese S, Croatto G, Monaco F, Krinitski D, Arrondo G, et al. Efficacy and acceptability of pharmacological, psychosocial, and brain stimulation interventions in children and adolescents with mental disorders: an umbrella review. World Psychiatry. 2021;20(2):244–75. 10.1002/wps.20881. 34002501 10.1002/wps.20881 PMC8129843

[31] Mohammed AAR, Mourad GM, Abdel-Fatah WO. Intervention program for enhancing self-esteem and self-efficacy of preparatory school students exposed to bullying behavior. Helwan Int J Nurs Res Pract. 2025;4(12):349–63. 10.21608/hijnrp.2025.424024.1449.

[32] Elsayed Mohammed -SalahES, Abd El-Moaty Mahmoud R. Effect of acceptance and commitment training program on social anxiety and self-esteem among adolescents with stuttering. Egypt J Health Care. 2025;16(2):1336–50. 10.21608/ejhc.2025.444560.

[33] Sharma S, Agarwala S. Self-esteem and collective self-esteem among adolescents: an interventional approach. Psychol Thought. 2015;8(1):105–13. 10.5964/psyct.v8i1.121.

[34] Doumit R, Kazandjian C, Militello LK. COPE for adolescent Syrian refugees in Lebanon: a brief cognitive-behavioral skill-building intervention to improve quality of life and promote positive mental health. Clin Nurs Res. 2020;29(4):226–34. 10.1177/1054773818808114. 30477312 10.1177/1054773818808114

[35] Torabi F, Rassouli M, Nourian M, Borumandnia N, Shirinabadi Farahani A, Nikseresht F. The effect of spiritual care on adolescents coping with cancer. Holist Nurs Pract. 2018;32(3):149–59. 10.1097/HNP.0000000000000263. 29642129 10.1097/HNP.0000000000000263

[36] Gammon J, Morgan-Samuel H. A study to ascertain the effect of structured student tutorial support on student stress, self-esteem and coping. Nurse Educ Pract. 2005;5(3):161–71. 10.1016/j.nepr.2004.09.003. 19038195 10.1016/j.nepr.2004.09.003

[37] Li W, Guo Y, Lai W, Wang W, Li X, Zhu L, et al. Reciprocal relationships between self-esteem, coping styles and anxiety symptoms among adolescents: between-person and within-person effects. Child Adolesc Psychiatry Ment Health. 2023;17(1):21. 10.1186/s13034-023-00564-4. 36755330 10.1186/s13034-023-00564-4 PMC9909938

[38] Qi L, Zhang H, Nie R, Du Y. Resilience promotes self-esteem in children and adolescents with hearing impairment: the mediating role of positive coping strategy. Front Psychol. 2024;15:1341215. 10.3389/fpsyg.2024.1341215. 39759409 10.3389/fpsyg.2024.1341215 PMC11698259

[39] Sáez RodríguezDJ, Ortigosa Quiles JM, Riquelme Marin A, Suriá Martínez R. Self-esteem and coping strategies in adolescent cancer patients during the period of illness and follow-up. Eur J Investig Health Psychol Educ. 2024;14(5):1128–39. 10.3390/ejihpe14050074. 10.3390/ejihpe14050074 PMC11120105 38785572

[40] Kyabatiibwa A. Self-esteem, depression and coping strategies among adolescents in Kampala City Authority [PhD thesis]. Kampala: Makerere University; 2023. Available from: http://hdl.handle.net/20.500.12281/16379.

